# Supplementary material for: E3 ubiquitin ligase SKP2 limits autophagosome formation during Staphylococcus aureus infection
Source: Front Cell Infect Microbiol. 2026 Feb 27;16:1749151. doi: 10.3389/fcimb.2026.1749151 (PMC12982375; doi:10.3389/fcimb.2026.1749151)
Supplement: Supplementary file 1 [file Table1.docx]

**Supplementary Figures**

**E3 ubiquitin ligase SKP2 limits autophagosome formation during *Staphylococcus aureus* infection**

Abhishek K. Singh^1^, Madina Baglanova^1^, Eylin Topfstedt^1^, Kristin Surmann^2^, Silva Holtfreter^3^, Leif Steil^2^, Uwe Völker^2^, Michael Lammers^4^, Barbara M. Bröker^3^, Karsten Becker^1^, Clemens Cammann^1#^, Ulrike Seifert^1#*^

^1^Friedrich Loeffler-Institute of Medical Microbiology, University Medicine Greifswald, Greifswald, Germany

^2^Department of Functional Genomics, Interfaculty Institute for Genetics and Functional Genomics, University Medicine Greifswald, Greifswald, Germany

^3^Institute of Immunology, University Medicine Greifswald, Greifswald, Germany

^4^Department of Synthetic and Structural Biochemistry, Institute of Biochemistry, University of Greifswald, Greifswald, Germany.

**^#^**these authors share last authorship

*** Correspondence:**Ulrike Seifert - ulrike.seifert@med.uni-greifswald.de





**Supplementary Figure 1. Increased abundance of SKP2 upon *S. aureus* infection in SAEC cells**

**(A)** SAEC cells were infected for the depicted time points with *S. aureus* USA300 (MOI 10) and SKP2 and IκBα abundance was analyzed by immunoblot compared to uninfected controls. **(B, C)** Densitometric analysis of SKP2 and IκBα normalized to β-actin, relative expression was calculated to the 2 h uninfected control, n = 3. **(D, E)** Cytokine secretion was assessed via ELISA for IL-6, n = 3 (D) and IL-8, n = 3 (E). **(F)** Cytotoxicity was monitored by determining extracellular LDH at 2 h, 3 h, and 5 h post-infection, n = 3. Data in B-F are presented as mean ± SD (*p < 0.05; **p < 0.01, students t-test).





**Supplementary Figure 2. Increased abundance of SKP2 upon *S. aureus* infection in THP-1 cells**

**(A)** THP-1 cells were infected with *S. aureus* USA300 (MOI 10) for the depicted time points. SKP2 and IκBα abundance was analyzed by immunoblot compared to uninfected controls. **(B, C)** Densitometric analysis of SKP2 and IκBα normalized to β-actin, relative expression was calculated to the 2 h uninfected control, n = 3. **(D)** Cytokine secretion was assessed via ELISA for IL-1β, n = 3. **(E)** Cytotoxicity was monitored by determining extracellular LDH at 2 h, 3 h, and 5 h post-infection, n=3. Data in B-E are presented as mean ± SD (*p < 0.05, **p < 0.01, ***p < 0.001, students t-test).





**Supplementary Figure 3. Intracellular bacterial survival and cell cycle analysis upon infection and SKP2 inhibition**

**(A)** SKP2 was immunoprecipitated from THP-1 cells infected with *S. aureus* USA300 (MOI 10) for 5 h and analyzed with anti-SKP2 and anti-acetyl lysine antibody compared to uninfected controls, heavy chain is indicated as unspecific band from the precipitating SKP2 antibody, n = 2. **(B)** A549 cells treated with SKP2-siRNA or non-targeted control (NTC) siRNA or with 20 µM SZL-P141 inhibitor (added 6 h prior infection) were infected with *S. aureus* HG001 (MOI 20) for 5 h. Intracellular bacterial burden was determined as CFU normalized to 1 × 10⁵ host cells, n = 3. **(C, D)** Cytokine secretion was assessed via ELISA for IL-6, n = 3 (C) and IL-8, n = 3 (D). **(E)** Flow cytometry analysis of the DNA content reflecting G1, G2 and S phase using Vybrant™ DyeCycle™ Violet stain. Statistical significance is indicated (*p<0.05, ****p<0.0001, students t-test).





**Supplementary Figure 4. SKP2 wildtype and mutant expression in HeLa cells**

**(A)** HeLa cells were transfected for 24 h with SKP2 (pcDNA SKP2), SKP2^K145R K228R^ (pcDNA SKP2mut) and empty vector control (pcDNA) or left untreated and analyzed for SKP2 expression by immunoblotting with β-actin as loading control. **(B)** HeLa cells were infected with *S. aureus* HG001 (MOI 10 and MOI 20) for the depicted time points and subsequently analyzed for SKP2 expression via immunoblotting with β-actin as loading control. **(C)** Densitometric analysis of SKP2 normalized to β-actin, relative expression was calculated to the 2 h uninfected control, n = 2.
